# Arcyriaflavin a, a cyclin D1–cyclin-dependent kinase4 inhibitor, induces apoptosis and inhibits proliferation of human endometriotic stromal cells: a potential therapeutic agent in endometriosis

**DOI:** 10.1186/s12958-017-0272-3

**Published:** 2017-07-18

**Authors:** Tomoko Hirakawa, Kaei Nasu, Yoko Aoyagi, Kanetoshi Takebayashi, Hisashi Narahara

**Affiliations:** 1 Department of Obstetrics and Gynecology, Faculty of Medicine, Oita University, Idaigaoka 1-1, Hasama-machi, Yufu-shi, Oita 879-5593 Japan; 2 Division of Obstetrics and Gynecology, Support System for Community Medicine, Faculty of Medicine, Oita University, Oita Prefecture, Oita 879-5593 Japan

**Keywords:** Endometriosis, Cyclin D1 inhibitor, Cell cycle, Apoptosis, Cell proliferation

## Abstract

**Background:**

We previously showed that microRNA-503 (miR-503) transfection into endometriotic cyst stromal cells (ECSCs) induced cell cycle arrest at the G0/G1 phase by suppressing cyclin D1. This finding prompted us to evaluate the potential therapeutic effects of cyclin D1 inhibitors in endometriotic cells. This study aimed to determine whether arcyriaflavin A, a representative inhibitor of cyclin D1–cyclin-dependent kinase 4 (CDK4), is beneficial in the treatment of endometriosis.

**Methods:**

ECSCs were isolated from the ovarian endometriotic tissues of 32 women. The effects of arcyriaflavin A on cell viability and proliferation, vascular endothelial growth factor A expression, apoptosis, and cell cycle progression were evaluated using a modified methylthiazoletetrazolium assay, enzyme-linked immunosorbent assay (ELISA), Caspase-Glo® 3/7 assay, and flow cytometry.

**Results:**

Arcyriaflavin A significantly inhibited cell viability, proliferation, and angiogenesis of ECSCs as assessed using the 5-bromo-2-deoxyuridine (BrdU) and methylthiazoletetrazolium bromide (MTT) assays, and vascular endothelial growth factor (VEGF) ELISA. Arcyriaflavin A induced apoptosis as shown in the Caspase-Glo® 3/7 assay and cell death detection ELISA whilethe cell cycle was arrested at the G0/G1 phase.

**Conclusion:**

The findings indicate that cyclin D1–CDK4 inhibitors may be promising candidates for the treatment of endometriosis. This is the first study to demonstrate the potential usefulness of arcyriaflavin A as a therapeutic agent for endometriosis. Further studies of the effects of cyclin D1–CDK4 inhibitors on endometriosis may provide useful information on pathogenesis and treatment.

## Background

Endometriosis is an estrogen-dependent condition characterized by the benign ectopic growth of proliferative endometrial tissue. It most frequently occurs in women of reproductive age and usually involves the peritoneum, ovaries, and rectovaginal septum [1]. Its main symptoms are dysmenorrhea, chronic pelvic pain, subfertility, and dyspareunia, which often greatly decrease the quality of life of the patients [1].

Although endometriotic tissue shares many histological characteristics with normal proliferative endometrial tissues [1], there are several interesting molecular differences such as in gene expression and protein production, synthesis, and responsiveness to steroids and cytokines. Endometriotic cells exhibit proliferative, antiapoptotic, angiogenic, and fibrogenic features [2–5].

To identify the mechanisms underlying the pathogenesis of endometriosis, our research has focused on the dysregulation of the expression of several microRNAs (miRNAs) involved in endometriosis [4–6]. miRNAs, which regulate the translation of specific targeted protein-coding genes, are short noncoding RNAs. In a previous study, we observed the anti-proliferative, pro-apoptotic, angiostatic, and anti-fibrogenic functions of miR-503 and identified its possible downstream targets using miR-503-transfected endometriotic cyst stromal cells (ECSCs) [5]. The transfection of miR-503 into ECSCs induces apoptosis by B-cell lymphoma-2 (Bcl-2) suppression, inhibition of vascular endothelial growth factor A (VEGF-A) production and cell proliferation, and induction of cell cycle arrest at the G0/G1 phase by cyclin D1 suppression [5]. These findings prompted us to evaluate the therapeutic effects of cyclin D1 inhibitors on endometriotic cells.

Numerous studies have reported the functions of cyclin D1 and cyclin-dependent kinase (CDK). Cyclin D1 interacts with the enzymes CDK 4/6 to regulate cell cycle progression from the G1 phase to the S phase [7]. There are few reports on the effects of cyclin D1–CDK4 inhibitors on endometriosis.

We hypothesized that cyclin D1 and CDK inhibitors might alleviat endometriosis. Arcyriaflavin A, a representative cyclin D1–CDK4 inhibitor, exhibited potent inhibition of cyclin D1–CDK4 [8]. Furthermore, it was previously described as a novel antiviral compound [9] and was reported to induce cancer cells apoptosis [10]. Therefore, we designed the present study to evaluate the efficacy of arcyriaflavin A in treating endometriosis.

## Methods

### Isolation of ECSCs and cell culture conditions

Ovarian endometriosis tissue_samples were obtained from patients with regular menstrual cycles who had undergone salpingo-oophorectomy or cystectomy for the treatment of ovarian endometriotic cysts (27 patients, aged 26–45 years). None of the patients had received any hormonal treatments for at least 6 months prior to surgery. All specimens were confirmed to_be in the mid- to late-proliferative phases based on pathological observation, the menstrual cycles, or both. The ECSCs were isolated from the ovarian endometriotic tissues using enzymatic digestion with collagenase, as previously described [11]. Isolated ECSCs were cultured in Dulbecco’s modified Eagle’s medium (DMEM) supplemented with 100 IU/mL penicillin, 50 mg/mL streptomycin, and 10% heat-inactivated fetal bovine serum (FBS, all obtained from Gibco-BRL, Gaithersburg, MD, USA) at 37 °C in air containing 5% CO_2_. The purity of ECSCs in the monolayer culture after the third passage was >99%, determined using immunocytochemical staining with antibodies for vimentin, CD10, cytokeratin, factor VIII, and leukocyte common antigen [11]. Each experiment was performed in triplicate and repeated with at least three replicates with cells from at least three different patients.

### Assessment of viability of arcyriaflavin A-treated cells

The viability of ECSCs after arcyriaflavin A treatment was determined using a modified methylthiazoletetrazolium (MTT) assay using the cell proliferation kit I (Roche Diagnostics GmbH, Basel, Switzerland). Briefly, 5 × 10^3^ ECSCs were placed into each well of 96-well flat-bottomed microplates (Corning, New York, NY, USA) and incubated with arcyriaflavin A (0.1–10 μM, diluted with culture medium, Santa Cruz Biotechnology, Dallas, TX, USA) for 48 h. Thereafter, 20 μL of the MTT dye was added to each well, the cells were incubated for 4 h, and then, the cell viability was determined by measuring the absorbance at 570 nm. The data were obtained from triplicate samples, and the values obtained from the arcyriaflavin A-treated ECSCs are presented as a percentage of the values of untreated ECSCs.

### Assessment of proliferation of arcyriaflavin A-treated ECSCs

The proliferation of ECSCs following arcyriaflavin A treatment was determined based on the 5-bromo-2′-deoxyuridine (BrdU) incorporation using an enzyme-linked immunosorbent assay (ELISA) kit (cell proliferation ELISA, Roche Diagnostics) [6]. We seeded 5 × 10^3^ ECSCs into each well of a 96-well flat-bottomed microplate (Corning), incubated them with arcyriaflavin A (0.1–10 μM) for 48 h, and then 10 μL BrdU (10 mM) was added to each well, followed by incubation for 2 h. The incorporation of BrdU was determined by measuring the absorbance of the resultant solution at 450 nm. The data were obtained from triplicate samples, and the values of the arcyriaflavin A-treated ECSCs are presented as a percentage of values of untreated ECSCs.

### Assessment of VEGF-A protein levels of arcyriaflavin A-treated ECSC culture supernatant

The protein levels of VEGF-A in the supernatant of ECSCs following arcyriaflavin A treatment were determined using an ELISA, as described previously [4]. Briefly, subconfluent ECSCs treated without and with arcyriaflavin A (0.1–10 μM) were cultured for 48 h in 24-well culture plates (Corning), and the supernatants were collected. The concentration of VEGF-A was determined using commercially available ELISA kits (Human VEGF immunoassay, R&D Systems, Minneapolis, MN, USA). The sensitivity of the VEGF-A assay was 9.0 pg/mL.

### Assessment of apoptosis of arcyriaflavin A-treated ECSCs

We determined the apoptosis levels of ECSCs following arcyriaflavin A treatment by direct determination of nucleosomal DNA fragmentation using an ELISA (cell death detection ELISA, Roche Diagnostics) [6]. The ECSCs (5 × 10^3^ cells/well) were placed in 96-well flat-bottomed microplates (Corning). After incubation with arcyriaflavin A (0.1–10 μM) for 48 h, the cells were lysed, centrifuged at 200×*g* for 10 min, and the mono- and oligo-nucleosomes in the supernatants were quantified using an anti-histone-biotin antibody. The concentration of the nucleosome-antibody complex was determined by measuring the absorbance at 405 nm using 2,2′-azino-di(3-ethylbenzthiazolinesulfonate) as the substrate. The data analyzed were from triplicate samples, and values of the arcyriaflavin A-treated ECSCs are presented as a percentage of those from untreated ECSCs.

### Assessment of caspase-3 and caspase-7 activities in arcyriaflavin a treated ECSC

The caspase-3 and caspase-7 activities of ECSCs following incubation with arcyriaflavin A were evaluated using the Caspase-Glo® 3/7 assay (Promega, Madison, WI, USA), as described previously [6]. The ECSCs (5 × 10^3^ cells/well) were plated in 96-well flat-bottomed microplates (Promega). After a 48-h incubation with arcyriaflavin A (0.1–10 μM), the Caspase-Glo® 3/7 reagent was added to each well, the plates were shaken gently for 120 min at 20–25 °C, and then the luminescence was measured using a plate-reading luminometer. The data analyzed were of triplicate samples, and the values of ECSCs treated with arcyriaflavin A are presented as a percentage of those of the untreated ECSCs.

### Assessment of cell cycle of arcyriaflavin A-treated ECSCs

The cell cycle of ECSCs following treatment with arcyriaflavin A was analyzed using flow cytometry, as previously described [5, 12]. Briefly, 72 h after arcyriaflavin A treatment (10 μM), the ECSCs were trypsinized, rinsed in phosphate-buffered saline, fixed in 70% ethanol, and then incubated for 30 min at 4 °C in the dark with a solution containing 5 μg/mL propidium iodide and 1 mg/mL RNase (Sigma-Aldrich, St. Louis, MO, USA). Flow cytometric analysis of the cell cycle was performed after propidium iodide staining using the CellFIT program (Becton-Dickinson, Franklin Lakes, NJ, USA), which analyzed the S-phase using a ModFit model.

### Statistical analysis

The data analyzed were of triplicate samples and are presented as a percentage relative to the corresponding control values as the mean ± standard deviation. The data were appropriately analyzed using the Bonferroni method and Student’s *t*-test using the SigmaPlot 11.2 (Systat Software, Chicago, IL, USA) while a *p* < 0.05 was considered significant.

## Results

### Suppression of ECSC viability and proliferation by arcyriaflavin a treatment

**Fig. 1:**
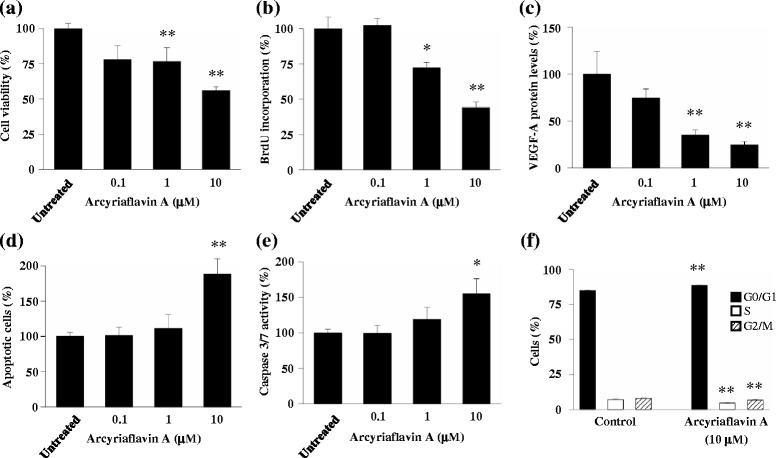
Therapeutic effects of arcyriaflavin A on endometriotic cyst stromal cells (ECSCs). **a** Cell viability; **b** 5-bromo-2-deoxyuridine (BrdU) incorporation; **c** vascular endothelial growth factor (VEGF)-A protein level; **d** apoptotic activity; **e** caspase-3/7 activity; **f** cell cycle progression. **a**–**e** ECSCs were analyzed following 48**-**h incubation with arcyriaflavin A. **f** ECSCs were analyzed using flow cytometry following a 72-h incubation with arcyriaflavin A. *p < 0.05 and **p < 0.005, Bonferroni method

The effects of arcyriaflavin A on the viability and proliferation of ECSCs were evaluated using modified MTT and BrdU incorporation assays, respectively. As shown in Fig. 1a, the number of viable cells decreased significantly after treatment with arcyriaflavin A at 1 and 10 μM. Furthermore, arcyriaflavin A treatment significantly inhibited BrdU incorporation in ECSCs at 1 and 10 μM (Fig. 1b).

### Downregulation of VEGF-A expression in ECSCs by arcyriaflavin a treatment

VEGF-A protein expression in ECSCs was suppressed by arcyriaflavin A at 1 and 10 μM (Fig. 1c).

### Induction of ECSC apoptosis by arcyriaflavin a treatment

The effects of arcyriaflavin A on apoptosis in ECSCs were determined using an ELISA kit. As shown in Fig. 1d, arcyriaflavin A induced apoptosis at 10 μM. The pro-apoptotic effects of arcyriaflavin A on ECSCs were also assessed by evaluating caspase-3 and caspase-7 activities, which were significantly at 10 μM (Fig. 1e).

### Induction of cell cycle arrest in ECSCs by arcyriaflavin a treatment

The effects of arcyriaflavin A on the cell cycle were determined using flow cytometry. As shown in Fig. 1f, arcyriaflavin A induced the accumulation of ECSCs in the G0/G1 phase (*p* = 0.000, Bonferroni method), with a concomitant decrease in the proportion of cells in the S and G2/M phases (*p* = 0.001 and *p* = 0.000, respectively; Bonferroni method).

## Discussion

In our previous study, we investigated the expression of miR-503 in ECSCs and normal endometrial stromal cells isolated from eutopic endometrial tissues. We evaluated the effects of miR-503 on the cellular functions of ECSCs and the mechanisms underlying the suppression of miR-503 expression in ECSCs. Transfection of ECSCs with miR-503 inhibited cell proliferation and VEGF-A production and induced apoptosis and G0/G1 cell cycle arrest in these cells [5]. Furthermore, we previously demonstrated that Bcl-2 and VEGF-A expression was upregulated in ECSCs, and this upregulation was responsible for the anti-apoptotic and angiogenic features of endometriosis [4, 13].

Based on these findings, we designed the present study to investigate the potential of arcyriaflavin A, a cyclin D1-CDK4 inhibitor, for treating endometriosis. Similar to the effects of miR-503, arcyriaflavin A inhibited cell proliferation and VEGF-A production, and induced apoptosis and G0/G1 cell cycle arrest in these cells mainly at 1 and 10 μM.

Cyclin-CDK complexes regulate the progression of cells through the cell cycle. Strong lines of evidence suggest the involvement of D-type cyclins in the G1 phase through an association with CDK4 and CDK6. Aberrations in CDKs and their regulators have been found in a large percentage of human tumors including melanoma, lymphoma, and carcinomas of the breast, lung, ovary, oropharynx, and colon [8]. Ovarian endometriotic cyst is an ovarian tumor and endometriosis is a well-established ovarian cancer risk factor. Thus, inhibitors of cyclin-CDK complexes may offer a broad range of therapeutic applications in endometriosis.

Over the last decade, many small molecules have been reported as CDK inhibitors. In particular, arcyriaflavin A has shown strong inhibitory activity (half-maximal inhibitory concentration [IC_50_] = 140 nM) against cyclin D1-CDK4. Interestingly, it also demonstrated significant selectivity towards several other kinases including cyclin B-CDK1, cyclin E-CDK2, protein kinase A (PKA), and protein kinase C (PKC) [8]. Furthermore, arcyriaflavin A has been described as a novel antiviral compound that selectively inhibits the replication of the human cytomegalovirus [9].

miR-503 has been demonstrated to downregulate cyclin D1 expression and induce G0/G1 phase cell cycle arrest in several cell types [5, 7, 14]. We confirmed that arcyriaflavin A induced cell cycle arrest in the G1 phase of ECSCs. Induction of apoptosis and inhibition of cell proliferation and angiogenesis follow cell cycle arrest. Arcyriaflavin A exhibited a variety of therapeutic effects on ECSCs that are similar to those induced by miR-503 transfection. A number of CDK 4/6 inhibitors have been evaluated for clinical use [15–19]. With the development of more effective and less toxic agents, cyclin D1 inhibitors could be used clinically for treating endometriosis in the future.

There are some limitations to the present study. First, the effects of arcyriaflavin A were evaluated only in ECSCs, but not in normal endometrial stromal cells. Another limitation is the study design. There is currently no established protocol for assessing the effects of arcyriaflavin A on endometriotic lesions in vivo.

## Conclusion

We demonstrated that the cyclin D1-CDK4 inhibitor, arcyriaflavin A, exerted therapeutic effects on ECSCs that are similar to those of miR-503, which is considered a promising candidate for the treatment of endometriosis. Future studies on the effects of cyclin D1-CDK4 inhibitors on endometriosis may provide useful information on the pathogenesis of this condition.

## Abbreviations

CDK: Cyclin-dependent kinase
DSBs: DNA double-strand breaks
ECSCs: Endometriotic cyst stromal cells
miRNAs: microRNAs
PKA: Protein kinase A
VEGF-A: Vascular endothelial growth factor A
